# The Impact of Different Nutritional Approaches on Body Composition in People Living with Obesity

**DOI:** 10.1007/s13679-025-00636-w

**Published:** 2025-05-17

**Authors:** Martina Galasso, Ludovica Verde, Luigi Barrea, Silvia Savastano, Annamaria Colao, Gema Frühbeck, Giovanna Muscogiuri

**Affiliations:** 1 Dipartimento Di Medicina Clinica E Chirurgia, Centro Italiano Per La Cura E Il Benessere del Paziente Con Obesità (C.I.B.O), Università Degli Studi Di Napoli Federico II, Via Sergio Pansini 5, 80131 Naples, Italy; 2 Department of Public Health, University of Naples Federico II, Via Sergio Pansini 5, 80131 Naples, Italy; 3 Department of Medicine, Division of Endocrinology, University of Arizona, Tucson, AZ USA; 4 Dipartimento Psicologia E Scienze Della Salute, Università Telematica Pegaso, Centro Direzionale Isola F2, Via Porzio, 80143 Naples, Italy; 5 Unità di Endocrinologia, Diabetologia e Andrologia, Dipartimento di Medicina Clinica e Chirurgia, Università Degli Studi Di Napoli Federico II, Via Sergio Pansini 5, 80131 Naples, Italy; 6 Cattedra Unesco “Educazione Alla Salute E Allo Sviluppo Sostenibile”, University Federico II, 80131 Naples, Italy; 7 Metabolic Research Laboratory, Cancer Center Clínica Universidad de Navarra (CCUN), Avda. Pío XII, 36, 31008 Pamplona, Spain; 8 CIBER Fisiopatología de La Obesidad y Nutrición (CIBEROBN), Instituto de Salud Carlos III, Pamplona, Spain; 9 Obesity and Adipobiology Group, Instituto de Investigación Sanitaria de Navarra (IdiSNA), Pamplona, Spain; 10 Department of Endocrinology & Nutrition, Cancer Center Clínica Universidad de Navarra (CCUN), Avda. Pío XII, 36, 31008 Pamplona, Spain

**Keywords:** Obesity, Body composition, Nutritional approaches, Personalized nutrition, Weight loss maintenance

## Abstract

**Purpose of Review:**

This narrative review aimed to provide an overview of the current evidence on the impact of various nutritional strategies on body composition in people living with obesity (PLwO), with particular attention to fat mass (FM), fat-free mass (FFM), and fat distribution.

**Recent Findings:**

Obesity is increasingly linked to cardiometabolic complications, yet common diagnostic metrics such as body mass index (BMI) do not capture changes in FM or FFM. Recent studies highlight the variable effects of different dietary interventions on body compartments. High-protein and ketogenic diets are associated with greater preservation of FFM and reductions in visceral adipose tissue (VAT), while the Mediterranean diet shows promise for long-term adherence and improvements in metabolic health. Intermittent fasting and time-restricted eating demonstrate efficacy in FM reduction but present mixed results regarding FFM retention and sustainability.

**Summary:**

Dietary strategies exert diverse effects on body composition in PLwO, underscoring the importance of tailoring interventions to individual metabolic profiles and health goals. Personalized nutrition approaches that prioritize the preservation of lean mass and reduction of VAT, along with sustainable adherence, are critical for optimizing obesity management beyond weight loss alone.

**Graphical Abstract:**

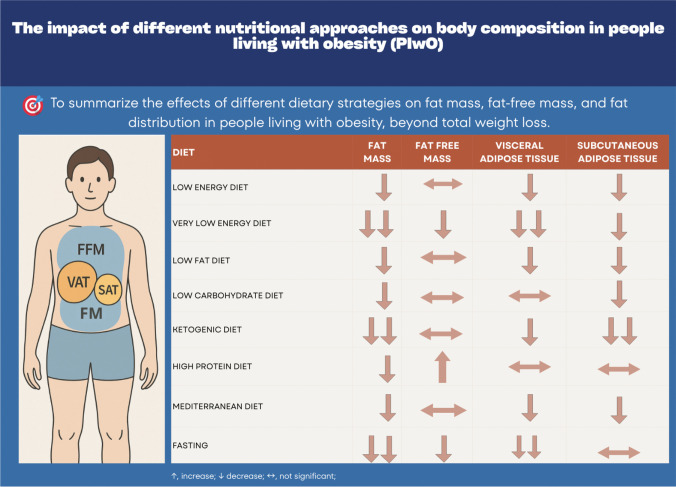

## Introduction

Obesity is a complex, multifactorial condition recognized as a major public health challenge worldwide due to its strong association with numerous chronic diseases, including type 2 diabetes (T2D), cardiovascular disease (CVD), metabolic dysfunction-associated steatotic liver disease (MASLD), and certain cancers [1]. Over the past few decades, the prevalence of obesity has risen sharply, with rates doubling in more than 70 countries since 1980 [2]. This surge has been attributed to a combination of factors such as excessive caloric intake, reduced physical activity, and an increasingly obesogenic environment [2, 3].

The conventional approach to diagnosing and classifying obesity has primarily relied on body mass index (BMI), a simple and cost-effective tool used globally [4]. However, BMI only provides a rough estimation of adiposity based on weight and height, failing to distinguish between fat mass (FM) and fat-free mass (FFM) [5, 6]. This limitation is particularly relevant when considering that obesity is fundamentally defined by an excess of adiposity rather than excess weight alone [7]. Therefore, BMI may not accurately reflect an individual’s cardiometabolic risk or response to interventions aimed at weight reduction [8].

Assessing body composition by differentiating between FM and FFM provides valuable insights into obesity-related health risks [9]. FM represents overall body fat, yet distinct fat depots exhibit different metabolic and health implications [9]. Specifically, visceral adipose tissue (VAT), located around internal organs, is strongly associated with metabolic complications, whereas subcutaneous adipose tissue (SAT), found beneath the skin, may have a more benign or even protective role [10]. Moreover, preserving or enhancing FFM during weight loss interventions is critical for metabolic health, physical function, and long-term weight maintenance [10].

Nutritional interventions employed in the treatment of obesity have varying impacts on body composition.

While caloric restriction is a common denominator and typically results in a reduction of FM, it can also lead to a loss of FFM [11]. Additionally, the manipulation of macronutrient composition can differentially influence changes in body compartments [11]. For example, high-protein diets may better preserve FFM, while very low-carbohydrate ketogenic diets often induce significant reductions in FM, particularly VAT [12]. Although body composition is increasingly recognized as a crucial factor in obesity management, it remains underemphasized in clinical practice, where weight loss is often prioritized over improvements in FM and FFM.

However, different nutritional strategies can have distinct effects on body compartments, influencing not only overall weight but also fat distribution and muscle preservation.

Research on dietary interventions for obesity has predominantly focused on weight loss outcomes, often relying on body mass index (BMI) as the primary metric. However, BMI alone does not provide an accurate assessment of changes in FM and FFM, which are critical for metabolic health and long-term weight maintenance [5].

Furthermore, variations in study methodologies, including differences in dietary protocols, duration, and body composition assessment techniques, contribute to inconsistencies in findings.

Given these considerations, this review aimed to analyze the impact of various nutritional approaches on body composition in people living with obesity (PLwO), highlighting their effects on FM, FFM, and fat distribution.

## Methods

This review employed a narrative methodology to synthesize and contextualize the existing evidence on the effects of various nutritional interventions on body composition in PLwO. The literature was primarily sourced through a structured search on the PubMed database, with additional searches conducted via Scopus and Web of Science to ensure a broader and more comprehensive inclusion of studies. Search terms included "obesity," "body composition," "fat mass," "fat-free mass," "visceral adipose tissue," "subcutaneous fat," "low-carbohydrate diet," "ketogenic diet," "high-protein diet," "Mediterranean diet," "intermittent fasting,"and"time-restricted eating." Only articles published in English were considered, with no restrictions on publication date, to capture both seminal and recent contributions to the field. Supplementary references were retrieved through manual screening of bibliographies from key reviews and original studies. Given the wide variability in dietary protocols and techniques used to assess body composition, the narrative review format was considered the most suitable for integrating and interpreting the data in a clinically relevant context.

In this review, the terms FFM and lean mass (LM) are used according to their conventional definitions [13]. FFM refers to all non-fat components of the body, including lean tissue, bone mineral content (BMC), and body water, while LM refers specifically to the fat-free soft tissues, excluding bone mass [13]. Whenever possible, the term used reflects the original terminology reported in the cited studies.

## Nutritional approaches and their effects on body composition

### Assessment of body composition

Accurate assessment of body composition is essential for evaluating the impact of different nutritional approaches in PLwO [9]. Several techniques are available to measure FM, FFM, and other relevant parameters [14].

Dual-energy X-ray absorptiometry (DEXA) is widely used for its precision in distinguishing fat distribution and lean mass [15]. Bioelectrical impedance analysis (BIA) offers a more accessible and cost-effective tool, though its reliability may be influenced by hydration status [16, 17]. Imaging techniques such as magnetic resonance imaging (MRI) and computed tomography (CT) provide detailed assessments of adipose tissue distribution but are less feasible in routine clinical settings due to higher costs and logistical demands [18].

Air displacement plethysmography (ADP), commonly referred to as the BOD POD, is another reliable method for estimating body composition and is often used in both clinical and research environments [19]. The ViScan, a specialized abdominal BIA device, enables distinction between visceral and subcutaneous adiposity and has demonstrated clinical relevance in assessing obesity-related risk [20]. Although skinfold measurements are still employed, their accuracy is limited by operator variability and reliance on prediction equations [21].

In practice, the choice of assessment technique should consider resource availability, clinical context, and the specific objectives of the evaluation—particularly in monitoring changes in fat mass and lean mass throughout dietary treatment.

### Caloric Restriction and Energy Deficit (LED, VLED)

Low-energy diets (LED) and very-low-energy diets (VLED) are structured nutritional interventions aimed at inducing significant weight loss through severe caloric restriction [23]. Table 1 shows the composition of LED and VLED. VLEDs are typically formulated as commercially prepared liquid products designed to replace all regular meals and are fortified with essential micronutrients to prevent deficiencies [23].

**Table 1:** Effects of nutritional approaches on body composition

Diets*	Composition of diet	FM	FFM	VAT	SAT
Low energy diet (LED) [22]	800–1200 kcal/die, P 15–25%, C 45–55%, F 20–30%	**↓**	** ↔ **	**↓**	**↓**
Very low energy diet (VLED) [23]	400- 800 kcal/die, P 20%, C 60%, F 20%	**↓↓**	**↓**	**↓↓**	**↓**
Low fat diet (LFD) and very low fat diet (VLFD) [24]	P 10–35%, C 45–70%, F 10–25%	**↓**	** ↔ **	**↓**	**↓**
Low carbohydrate diet (LCD) [25]	P 20–30%, C 20–45%, F 30–50%	**↓**	** ↔ **	** ↔ **	**↓**
Ketogenic diet (KD) [26]	650–800 kcal/die, P 40–45%, C < 10%, F 30–40%	**↓↓**	** ↔ **	**↓**	**↓↓**
High protein diet (HPD) [27]	P > 25–35%, C 30–40%, F 25–35%	**↓**	↑	** ↔ **	** ↔ **
Mediterranean diet (MD) [28]	P 10–20%, F < 30%, C 45–55%	**↓**	** ↔ **	**↓**	**↓**
Fasting [29]	P 15–20% C 40–55%, F 20–30%	**↓↓**	**↓**	**↓↓**	** ↔ **

*with energy deficit. P, protein; F, fat; C, carbohydrate; FM, fat mass; FFM, fat free mass; VAT, visceral adipose tissue; SAT, subcutaneous adipose tissue; ↓ decrease; ↑ increase; ↔ non significant

The primary goal of VLEDs is to achieve rapid weight loss, approximately 1.0–2.5 kg per week, while preserving LM as much as possible [30]. However, even when protein intake is as low as 50 g/day, studies have reported that approximately 25% of total weight loss consists of LM, while 75% is fat loss [31].

As for body composition, studies showed that they were effective in inducing substantial short-term weight loss. For example, a randomized controlled trial (RCT) conducted by Harshal et al. compared VLEDs to moderate energy-deficit approaches in women living with obesity and polycystic ovary syndrome (PCOS), and body composition was measured at baseline and follow-up visits by DEXA [32]. After eight weeks, the VLED group exhibited significantly greater weight loss and reductions in WC and total and trunk fat compared to the conventional approach. However, both interventions resulted in losses of LM and FFM, while BMC and bone mineral density (BMD) remained unchanged [32].

Additionally, an observational prospective study by Serafim et al. evaluated body composition by BIA in 120 PLwO undergoing bariatric surgery (BMI: men 50.6 ± 1.12 kg/m^2^, women 49.1 ± 0.58 kg/m^2^) during a VLED [33]. The study reported significant reductions in weight and BMI, along with decreases in WC and hip circumference. Both FM and FFM showed significant reductions [33].

While aggressive caloric restriction can be an effective strategy for inducing rapid weight loss, its long-term effectiveness remains controversial. Meta-analyses have shown that VLEDs do not necessarily result in superior long-term weight loss compared to less restrictive approaches like LED [22]. Moreover, an RCT involving 49 women living with obesity compared long-term weight loss outcomes between a 1200 kcal/d diet of conventional foods and a VLED [34]. Women on the conventional diet, who lost an average of 11.9 kg over 6 months and attended 39 group behavioral maintenance sessions, maintained a loss of 12.2 kg after one year. Conversely, those who followed the VLED initially lost 21.5 kg over the same period but retained only 10.9 kg of that weight loss despite receiving the same maintenance sessions [34].

The effectiveness of VLEDs and LEDs in promoting weight loss and altering body composition is primarily driven by creating a substantial caloric deficit, resulting in negative energy balance [30]. This deficit forces the body to mobilize energy stores, particularly adipose tissue, to meet energy demands [30]. The significant reduction in carbohydrate intake associated with VLEDs induces glycogen depletion, which is accompanied by a loss of water weight, thereby contributing to rapid initial weight loss [22]. Additionally, the reduction in insulin levels and enhancement of lipolysis facilitate fat oxidation [22].

Despite their effectiveness in promoting rapid weight loss, VLEDs present several drawbacks that may compromise their long-term success and safety.

Excessive caloric restriction, especially when protein intake is inadequate, can lead to substantial loss of LM, negatively affecting metabolism and physical function [31]. Furthermore, the rapid weight loss associated with VLEDs can increase the risk of gallstone formation and exacerbate pre-existing medical conditions if not properly supervised [35]. Another significant limitation is the difficulty in maintaining weight loss over the long term.

Studies suggest that individuals following VLEDs may regain a substantial portion of lost weight unless accompanied by structured maintenance programs and behavioral interventions [34]. Additionally, adverse effects such as fatigue, dizziness, muscle cramps, constipation, and hair loss have been commonly reported during prolonged VLED use [22]. Continued research is necessary to identify strategies that minimize adverse effects while preserving LM and promoting sustainable weight loss.

### Low-Fat Diets

The composition of the low-fat diet (LFD) and very low-fat diet (VLFD) is reported in Table 1.

As for body composition, a systematic review by Hooper et al. examined 32 RCT involving approximately 54,000 PLwO [36]. The analysis demonstrated that reducing dietary fat, without intentional caloric restriction, consistently resulted in modest reductions in body weight, FM, and WC. However, the reduction in fat intake likely caused a reduction in total energy intake, indirectly contributing to weight loss [36].

VLFD has been primarily studied in the context of vegetarian and vegan diets [24]. Indeed, a recent RCT involving 168 adults aged 25 to 75 years with a BMI between 28 and 40 kg/m^2^ compared the effects of a low-fat vegan diet to a control diet [37]. Body composition was assessed using DEXA, revealing that the vegan group experienced significant reductions in body weight, primarily due to a decrease in FM and VAT.

While these diets have shown positive effects on weight loss, studies that include body composition analysis are limited.

Overall, while LFDs can produce modest weight loss and fat reduction, their impact on LM preservation remains unclear [37].

An RCT involving 148 PLwO compared a low-fat diet to a low-carbohydrate diet (with carbohydrate intake below 40 g per day), and body composition was measured using BIA [38]. Results indicated that adherence to the low-carbohydrate diet was associated with greater reductions in body weight and FM and, notably, an increase in FFM percentage. Specifically, higher adherence to the low-carbohydrate diet corresponded to more weight loss, FM reduction, and improved FFM preservation [38].

The rationale behind LFDs is that reducing dietary fat, the most energy-dense macronutrient, helps to create a caloric deficit [39]. Experimental studies manipulating fat content in diets have shown that higher-fat diets lead to greater weight gain or less weight loss due to their higher energy density [39].

However, the long-term effectiveness of LFDs remains unclear, as participants often adapt to the reduced energy density over time, potentially diminishing their impact on weight loss.

### Low-Carbohydrate Diets and Ketogenic Diets

The composition of the low carbohydrate diet (LCD) and ketogenic diet (KD) is reported in Table 1.

In recent years, KDs have attracted great interest in the treatment of PLwO and obesity-related metabolic disorders. However, although KDs are a dietary intervention designed to induce nutritional ketosis, different diets with different macronutirients compositions have been called this name, and this may result in bias and mistakes in the interpretation of current evidence. To clarify this variability, Table 2 also reports the main types of ketogenic dietary protocols currently used in clinical and research settings, highlighting their distinguishing macronutrient profiles. For PLwO, very low-calorie ketogenic diets (VLCKD), recently renamed very low energy ketogenic therapy (VLEKT) to avoid misconceptions with very low carbohydrate diets[40] are currently the most used in clinical practice. Of note, this definition emphasizes a more precise approach, characterized by both a minimal carbohydrate intake (< 50 g/day) and a significantly reduced caloric intake (typically under 800 kcal/day), which distinguishes it from other dietary approaches and better reflects its therapeutic application [40].

**Table 2:** Ketogenic protocols

Ketogenic protocols	Definition
Very low-energy ketogenic therapy (VLEKT)*	< 30–50 g/day of carbohydrates, < 700–800 kcal/day, < 30–40 g/day of fat
Low-calorie ketogenic diet (LCKD)	< 30–50 g/day of carbohydrates, > 700–800 kcal/day and < TEE, > 30–40 g/day of fat
Isocaloric ketogenic diet (ICKD)	< 30–50 g/day of carbohydrates, in line with total energy expenditure, 70–80% of daily calorie intake from fat
ATKINS diet	progresses from a very low-carb, high-fat phase (≤ 20 g carbs/day) to a more flexible maintenance phase (80–100 g carbs/day), with protein intake remaining moderate throughout. Fat intake is high in early phases and moderates as carb intake increases

Notes: * Recently renamed, previously called very low-calorie ketogenic diet (VLCKD)

The European guidelines for managing PLwO have recently shown the effects of VLEKT on body weight and body composition [26]. In the short, medium, and long terms, the authors found that VLEKT caused a notable reduction in body weight. Additionally, VLEKT has shown greater success in reducing body weight, namely WC and FM, when compared to other weight loss dietary treatments of the same length. Crucially, FFM preservation and the selective burning of fat in visceral rather than subcutaneous adipose tissue compartments optimize body composition and result in weight loss during VLEKT [26].

Furthermore, VLEKT, has shown greater reductions in FM compared to the Western diet [41]. Paoli et al., in an RCT prospective study of 16 athletes of normal weight, found a significantly higher decrease of FM (p = 0.0359), VAT (p = 0.0018), WC (p = 0.0185), and extracellular water (p = 0.0060) in the KD compared to the WD group. Body composition was assessed using DEXA. Lean soft tissue, quadriceps muscle area, maximal strength, and REE (resting energy expenditure) showed no changes in both groups [41]. The mechanisms underlying the potential benefits of KD and LCD on body composition remain debated.

KD is proposed to enhance fat oxidation through reduced insulin levels, promoting greater fat loss [42]. Additionally, KD appears to offer notable benefits in appetite regulation and spontaneous reduction in energy intake, particularly under ad libitum conditions [43–47]. This appetite-suppressing effect may contribute to fat loss without purposeful caloric restriction, potentially making KD a valuable tool for weight management [43–47]. However, rigorous studies comparing isocaloric, protein-matched KD to non-KD conditions have not demonstrated significant metabolic advantages in fat loss, suggesting that the greater weight loss observed with KD may be largely attributed to higher protein intake, which enhances satiety and reduces overall energy intake [48]. Furthermore, a comprehensive review by Hall and Guo, including 32 isocaloric, protein-matched studies, found no evidence supporting a metabolic advantage of carbohydrate restriction [49].

While LCDs and KDs are effective in reducing body weight and FM, their ability to preserve or enhance LM remains inconsistent. Future research should focus on distinguishing whether the benefits are primarily due to macronutrient composition or overall caloric reduction. Nonetheless, KD may hold specific advantages in terms of appetite control and adherence, making it a promising dietary approach for weight and FM management.

### High-Protein Diets

High-protein diets (HPDs) lack a universally accepted definition, but they are generally described as providing at least 25% of total energy from protein [24]. Table 1 shows the composition of HPDs. As for body composition, research conducted by Layman et al. demonstrated that consuming protein at twice the RDA (1.6 g/kg) consistently outperformed the standard RDA (0.8 g/kg) in preserving LM and reducing FM [50, 51]. However, recent studies by Longland et al. have shown that, under conditions involving high-intensity interval training and resistance exercise, higher protein intake (2.4 g/kg) resulted in gains in LM and substantial FM. In contrast, a lower intake (1.2 g/kg) only managed to preserve LM and led to less FM [52].

Arciero et al. further supported these findings through their studies on “protein-pacing” strategy involving the consumption of 4–6 meals per day with over 30% of protein per meal (equating to > 1.4 g/kg/d). This approach has demonstrated superior outcomes in improving body composition compared to traditional lower-protein diets under hypocaloric conditions for FM, central adiposity, and increases in LM [53, 54].

Protein is known for its high thermic effect and considerable metabolic cost. Enhanced protein intake has been associated with the preservation of resting energy expenditure during caloric restriction [27].

### Mediterranean Diet and Quality-Based Approaches

The composition of macronutrients in the Mediterranean diet (MD) is reported in Table 1.

The MD is recognized as a promising nutritional strategy for managing weight and body composition [9]. Its main characteristics include a high intake of vegetables, fruits, nuts, whole grains, and extra-virgin olive oil, with moderate consumption of fish and poultry, while limiting sweets, red meat, and dairy products [55]. This dietary pattern is rich in monounsaturated fats (MUFA) and fiber, with low levels of saturated fats and a balanced omega-6/omega-3 ratio [56]. Interventional studies suggest that the effects of the MD on body weight are more closely related to energy intake than macronutrient composition. Notably, even when calorie intake is not restricted, this dietary pattern does not lead to weight gain [57].

The largest RCT to date involved 7,447 participants (most of whom with overweight or obesity), divided into three groups: MD supplemented with olive oil, MD supplemented with nuts, and a low-fat diet. The median follow-up period was 4.8 years [58]. In the MD groups, fats accounted for approximately 42% of daily energy intake, with no calorie restriction or promotion of physical activity, despite a high prevalence of overweight and obesity.At the end of the study, all groups exhibited slight weight reductions but increased WC. Compared to the low-fat diet group, neither of the MD groups showed significant differences in body weight. However, the MD was associated with reduced central adiposity, as reflected by adjusted WC differences after 5 years [58].

A meta-analysis of 16 RCTs (*n* = 3,436) found that the MD was associated with greater weight loss compared to control diets, particularly when combined with calorie restriction and increased physical activity [59]. Another meta-analysis of five RCTs (n = 998) reported that the MD was more effective than low-fat diets for weight loss but showed similar results to low-carbohydrate and the American Diabetes Association diets. Effects on BMI and WC were comparable to those on weight reduction [60].

Cross-sectional studies have also reported an inverse association between adherence to the MD and central adiposity [61–63]. The positive effects on central adiposity and VAT are attributed to high MUFA and polyunsaturated fatty acid (PUFA) intake and reduced saturated fat intake [56].

Short-term studies demonstrated that the isocaloric MD rich in extra-virgin olive oil prevents central fat accumulation better than low-fat diets without significantly affecting body weight in PLwO [64]. Other interventions reported reduced VAT with the MD over 2 months [65, 66]. A protein-enriched, calorie-restricted MD for eight weeks resulted in substantial reductions in weight, VAT, and FM, while preserving FFM in PLwO awaiting bariatric surgery [66].

A systematic review of 18 trials involving 7,186 PLwO concluded that the MD reduces central adiposity as shown by decreased WC, waist-hip ratio, and VAT, although the most consistent results were seen for WC [67].

Overall, the MD appears to be effective in reducing body weight, particularly when energy-restricted and combined with exercise. Even when not energy-restricted, it does not promote weight gain. The MD also demonstrates potential for reducing central adiposity and metabolically harmful VAT, making it a beneficial choice for PLwO or those at risk of cardiovascular and metabolic diseases.

### Fasting and Time-Restricted Eating

The composition of macronutrients during fasting is reported in Table 1. Fasting encompasses various dietary protocols (Table 3) aimed at cycling between periods of eating and fasting to achieve caloric restriction and metabolic benefits [68]. The primary approaches to fasting can be categorized into alternate-day fasting (ADF), whole-day fasting (WDF), intermittent fasting (IF), and time-restricted feeding (TRF) [68].

**Table 3:** Fasting protocols

Fasting protocols	Definition
Involves ≥ 24 Hours of Continuous Fasting	
Alternate-day fasting	Fasting (24-h caloric fast with < 30% of normal caloric intake) every other day or every three to five days, with unrestricted eating on intermittent days
Short-term fasting	Calorie-restricted fasting for one or more days, typically before and after pharmacological treatment/chemotherapy, with a normocaloric diet between cycles
Involves < 24 Hours of Continuous Fasting	
Time-restricted eating	Eating is restricted within a time window of 4 to 12 h each day, with fasting for the remainder of the 24-h period
Prolonged overnight fasting	A type of TRE involving fasting during nighttime hours and unrestricted eating during daylight hours
Ramadan fasting	During the month of Ramadan, some Muslim individuals fast during daylight hours and eat only before dawn or after dusk
Fasting-mimicking diet	A calorie-restricted diet (10% to 50% of normal caloric intake) designed to mimic the fasting state. Typically administered for multiple days before and 24 h after pharmacological treatment, often using plant-based foods

TRE, time-restricted eating.

ADF is one of the most extensively studied forms of fasting [69]. It involves a 24-h fasting period alternated with a 24-h ad libitum feeding period [69]. Research indicates that total energy intake tends to be reduced on feeding days, leading to weight loss and FM reduction even without deliberate caloric restriction [70]. ADF also shows potential for preserving LM, although some studies have reported LM loss due to severe energy deficits. Notably, Catenacci et al., in a RCT in 45 PLwO (BMI ≥ 30 kg/m^2^, aged 18–55 years) and evaluated body composition by DEXA and RMR [71] found that ADF, involving zero caloric intake on fasting days alternating with unrestricted feeding days, produced comparable results to daily caloric restriction in terms of body composition, in particular a significant reduction in total FM and trunk FM after 8 weeks of intervention, and even demonstrated superior outcomes after six months of unsupervised weight loss maintenance [71].

A variant of ADF, known as alternate-week energy restriction, involves one week of approximately 1300 kcal/day followed by a week of usual diet [72]. This approach has proven as effective as continuous energy restriction in reducing body weight and WC over both short-term (8 weeks) and long-term (1 year) periods [72].

TRF typically involves limiting food intake to specific periods each day, generally ranging from 16 to 20 h of fasting, followed by a feeding window of 4 to 8 h [73]. One of the most studied forms of TRF is Ramadan fasting, which involves refraining from eating and drinking from sunrise to sunset for approximately one month, resulting in weight loss through reductions in both LM and FM [74].

More structured studies on TRF include an 8-week study by Tinsley et al., which applied a protocol of 20 h of fasting followed by a 4-h feeding window, four days per week, in healthy, recreationally active men and assessed body composition by DEXA [75]. Despite a significant reduction in caloric intake on fasting days, muscle growth was similar in both the TRF and normal diet groups when combined with resistance training. However, the TRF group exhibited a slight tendency toward LM loss, although strength improvements were comparable between groups [75].

In contrast, Moro et al. investigated a 16-h fasting and 8-h feeding protocol in resistance-trained participants and evaluated the body composition by DEXA [76]. Their findings demonstrated significantly greater FM loss in the TRF group compared to those following a normal diet, with no significant impact on LM. Enhanced fat loss was suggested to be linked to elevated adiponectin levels, which may promote mitochondrial biogenesis and enhance energy expenditure. However, the TRF group also experienced unfavorable hormonal changes, such as reduced testosterone and triiodothyronine levels [76].

Furthermore, Deying et al. [29] conducted a 12-month study involving 139 PLwO (BMI 28–45 kg/m^2^), randomly allocated to either a TRF (8:00 a.m. to 4:00 p.m.) with calorie restriction or daily calorie restriction without time limitation, and evaluated body composition by DEXA. All participants adhered to a calorie-restricted diet, and after 12 months, the average weight loss was − 8.0 kg in the TRF group and − 6.3 kg in the daily calorie restriction group. The difference in weight loss between the groups was not statistically significant. Additional analyses of WC, BMI, BF, LM, blood pressure, and metabolic risk factors produced results consistent with the primary outcome, with no significant differences in the occurrence of adverse events between the two groups [29].

## Clinical and Practical Considerations

### Long-Term Adherence and Sustainability

Sustainability is a critical factor in dietary success and plays a fundamental role in long-term weight management [77]. Importantly, not only is achieving weight loss a goal, but maintaining the weight loss over time is equally, if not more, crucial for improving health outcomes and preventing weight cycling [77]. Restrictive diets, such as VLEDs and KDs, may lead to significant short-term weight loss and metabolic benefits but are often difficult to maintain due to their rigid structure, potential nutrient deficiencies, and social limitations [34]. Studies indicate that adherence rates tend to decline over time, with many individuals experiencing weight regain once the diet is discontinued [77]. In contrast, dietary approaches emphasizing flexibility, variety, and quality, such as the MD, tend to show higher adherence rates due to their palatability, nutritional adequacy, and adaptability to different cultural and individual preferences [78]. Furthermore, behavioral, and psychological factors significantly influence long-term adherence, highlighting the need for strategies that incorporate dietary education, gradual modifications, and personalized support to enhance sustainability.

### Risks and Benefits of Different Dietary Strategies

Each dietary approach presents unique risks and benefits that must be carefully weighed to optimize health outcomes. VLEDs and LCDs facilitate rapid weight loss by creating a substantial energy deficit, but they may compromise LM if not adequately designed with sufficient protein intake and resistance training [31]. Additionally, these diets can increase the risk of gallstone formation, metabolic adaptation, and micronutrient deficiencies if not properly supervised [35]. KD enhances fat oxidation and has demonstrated efficacy in reducing VAT, but it may lead to potential micronutrient deficiencies due to the restriction of certain food groups, as well as adverse effects such as fatigue, gastrointestinal distress, and potential long-term cardiovascular risks if not properly balanced [42]. High-protein diets support LM preservation and can improve satiety, thermogenesis, and overall metabolic health, but they require careful monitoring to avoid excessive intake of processed protein sources, which may contribute to renal strain, altered gut microbiota composition, and increased saturated fat intake [27]. Personalized dietary modifications and professional supervision can help mitigate these risks while optimizing the benefits of each approach for individual needs.

### Personalization Based on Patient Characteristics

Dietary strategies should be tailored to individual metabolic responses, comorbidities, and lifestyle preferences (see Table 4). Factors such as age, sex, gut microbiota composition, and physical activity levels can influence dietary needs and responses to different nutritional interventions [79]. Notably, PLwO who present obesity-related complications, such as T2D, dyslipidemia, and CVD, may benefit from more targeted dietary approaches, as these conditions not only affect metabolic regulation but also modify how different diets influence body composition. For instance, individuals with T2D may respond more favorably to lower-carbohydrate or moderate-carbohydrate diets with a low glycemic index, which can improve glycemic control and reduce visceral adiposity [80]. In cases of dyslipidemia, dietary patterns rich in unsaturated fats, such as the MD, have been shown to improve lipid profiles while supporting fat mass reduction and lean mass preservation. Similarly, in patients with CVD or high cardiovascular risk, dietary interventions that emphasize anti-inflammatory and cardioprotective properties—such as increased intake of omega-3 fatty acids, fiber, and antioxidants—may be more appropriate and beneficial [81]. These nuances highlight the importance of tailoring nutritional strategies not only to support weight loss and lean mass preservation but also to address disease-specific pathophysiological mechanisms, ultimately enhancing clinical outcomes [82]. Precision nutrition approaches integrating genetic, metabolic, and behavioral data may further improve dietary efficacy and adherence, offering more individualized and effective interventions for this heterogeneous population [83].

**Table 4:** Summary of the main benefits (pros) and limitations (cons) of different diets used for weight management

Diets	Pros	Cons
Very low energy diet (VLED) and low energy diet (LED)	- Rapid weight loss and metabolic improvement	- Difficult to maintain long-term adherence - Potential nutrient deficiencies if not well planned - Social limitations - Potential fatigue
Low carbohydrates diet (LCD)	- Rapid weight loss and metabolic improvement	- Difficult to maintain long-term adherence - Potential nutrient deficiencies if not well planned - Social limitations - Potential fatigue
Ketogenic diet (KD)	- Rapid weight loss and metabolic improvement - Enhances satiety - Euphoric effect	- Difficult to maintain long-term adherence - Potential nutrient deficiencies if not well planned - Social limitations - Potential fatigue - Not suitable for everyone
Low fat diet (LFD) and very low fat diet (VLFD)	- May reduce overall calorie intake	- Potential nutrient deficiencies if not well planned
High protein diet (HPD)	- Enhances satiety	- Promote excess intake of processed proteins - Higher cost and reduced accessibility - Difficult adherence in some cultural or social contexts - Risk of dietary monotony
Mediterranean diet (MD)	- High long-term adherence - Nutritionally adequate - Adaptable to cultural preferences - Suitable for everyone	- Slower weight loss when not combined with exercise
Fasting (es. intermittent, time-restricted)	- Improves circadian alignment - Does not necessarily restrict food choices	- Hunger, irritability, fatigue, especially at the beginning - Social limitations - Not suitable for everyone

## Conclusions and Future Directions

### Summary of Available Evidence

Different nutritional approaches exhibit varying effects on body composition, with high-protein diets and KD showing superior LM preservation by enhancing muscle retention and promoting fat oxidation [42, 52]. MD provides a sustainable model for long-term weight management due to its balanced nutrient profile and high adherence rates [67]. IF and TRE offer alternative strategies for improving metabolic health, with evidence suggesting potential benefits in insulin sensitivity, FM reduction, and appetite regulation [74]. However, individual responses to these dietary strategies can vary, underscoring the importance of personalized nutrition plans tailored to metabolic needs and lifestyle preferences.

Moreover, achieving weight loss is only one part of the therapeutic goal; long-term weight maintenance is equally critical for sustaining metabolic benefits and preventing weight cycling, which has been associated with negative health outcomes, including lean mass loss, fat redistribution, and increased cardiometabolic risk [84].

Dietary approaches should therefore be evaluated not only for their efficacy in inducing initial weight loss but also for their capacity to support its maintenance over time [85].

### Need for Further Studies on Specific Approaches and Patient Subgroups

Future research should focus on optimizing dietary interventions for specific populations, including those with metabolic disorders, sarcopenic obesity, and weight cycling history. Additionally, investigating the impact of these interventions on hormonal regulation, gut microbiota, and inflammatory markers could provide deeper insights into the mechanisms underlying their effects. Long-term comparative studies assessing adherence, sustainability, and metabolic outcomes are needed, with a particular emphasis on real-world applicability and integration into routine clinical practice. Moreover, exploring the role of digital health tools and behavioral interventions in improving adherence and personalized dietary modifications will be crucial for enhancing long-term success.

### Implications for Clinical Practice and Future Research

Integrating body composition analysis into obesity management can enhance treatment efficacy.

Preserving lean mass should be recognized as a key therapeutic target due to its role in maintaining metabolic health, physical function, and long-term weight stability. As such, the routine clinical assessment of body composition—using reliable and accessible tools—should be considered an essential component of personalized obesity care, guiding both dietary strategies and follow-up interventions.

Clinicians should consider dietary quality, individual preferences, and metabolic responses when recommending nutritional interventions. Particular attention should be given to strategies that promote long-term weight maintenance, as this remains a significant challenge in clinical practice and a key determinant of sustained health improvement. Further research should explore personalized dietary approaches to improve long-term health outcomes in PLwO, with a focus on maintaining weight loss, preserving LM, and minimizing fat regain.

## Key References

- Powell-Wiley, T.M., et al., Obesity and Cardiovascular Disease: A Scientific Statement From the American Heart Association. Circulation, 2021. 143(21): p. e984-e1010.
  o Comprehensive overview of the pathophysiological links between obesity and cardiovascular disease.
- Salmon-Gomez, L., et al., Relevance of body composition in phenotyping the obesities. Rev Endocr Metab Disord, 2023. 24(5): p. 809–823.
  o Highlights the role of fat mass and fat-free mass in differentiating obesity phenotypes and associated risks.
- Holmes, C.J. and S.B. Racette, The Utility of Body Composition Assessment in Nutrition and Clinical Practice: An Overview of Current Methodology. Nutrients, 2021. 13(8).
  o Describes and compares body composition assessment techniques such as DXA, BIA, and MRI in clinical nutrition.
- Ardavani, A., et al., The Effects of Very Low Energy Diets and Low Energy Diets with Exercise Training on Skeletal Muscle Mass: A Narrative Review. Adv Ther, 2021. 38(1): p. 149–163.
  o Reviews the impact of caloric restriction and physical activity on muscle preservation during weight loss.
- Muscogiuri, G., et al., European Guidelines for Obesity Management in Adults with a Very Low-Calorie Ketogenic Diet: A Systematic Review and Meta-Analysis. Obes Facts, 2021. 14(2): p. 222–245.
  o Provides evidence-based guidelines supporting the use of very low-calorie ketogenic diets in obesity treatment.
- Liu, D., et al., Calorie Restriction with or without Time-Restricted Eating in Weight Loss. N Engl J Med, 2022. 386(16): p. 1495–1504.
  o A 12-month RCT comparing calorie restriction with and without time-restricted eating; no significant difference in weight loss or body composition outcomes.

## Data Availability

No datasets were generated or analysed during the current study.
